# Changes in late-life systolic blood pressure and all-cause mortality among oldest-old people in China: the chinese longitudinal healthy longevity survey

**DOI:** 10.1186/s12877-021-02492-4

**Published:** 2021-10-18

**Authors:** Hui Gao, Kan Wang, Fariba Ahmadizar, Wensui Zhao, Yu Jiang, Lei Zhang, Li Yu, Fangjia Zhou, Jialing Gu, Jianlin Zhuang, Zhao-lin Xia

**Affiliations:** 1 Changning Center for Disease Control and Prevention, 39 Yunwushan Road, P.O. Box1003, 200032 Shanghai, China; 2 Department of Epidemiology, Erasmus Medical Center, Rotterdam, the Netherlands; 3 School of Public Health, Fudan University, Shanghai, China

**Keywords:** Blood pressure, Blood pressure variation, Mortality, Oldest-old

## Abstract

**Background:**

Blood pressure targets for oldest-old people have been long debated due to the concern that more stringent targets are associated with increased mortality. We aimed to investigate the association between changes of late-life systolic blood pressure (SBP), mean SBP and SBP variability (SBPV), and all-cause mortality in oldest-old.

**Methods:**

Based on the community-based Chinese Longitudinal Healthy Longevity Survey with follow-up conducted in the 3-year interval, we assembled a retrospective cohort of 6639 participants ≥ 80 years with available blood pressure measurements at baseline and second wave. The primary exposures were mean SBP and SBPV (defined as the annual difference in SBP divided by mean SBP) measured between baseline and second wave. The primary outcome was all-cause mortality assessed from the second wave.

**Results:**

During 21443.1 person-years of follow-up, 4622 death was recorded. U-shaped associations of mortality with mean SBP and SBPV were identified; the value of 137 mmHg and 4.0 %/year conferred the minimum mortality risk, respectively. The associations of a larger SBPV with an increased mortality risk were observed for both rises and large falls in SBP. The hazard ratio was 1.11 (comparing lowest versus middle quintile; 95 % CI: 1.01, 1.22) with large falls in SBPV and 1.08 (comparing highest versus middle quintile; 95 % CI: 0.98, 1.18) with large rises in SBPV.

**Conclusions:**

U-shaped associations between late-life SBP and SBPV and all-cause mortality were found. Our study suggests that a stable SBP level in the middle range is related to lower mortality risk in the oldest-old.

**Supplementary Information:**

The online version contains supplementary material available at 10.1186/s12877-021-02492-4.

## Background

Hypertension is the leading determinant of major cardiovascular disease events and mortality, affecting over half of elderly people worldwide [1]. Its management is still far from ideal, especially in developing countries [2]. A key challenge for clinicians to enhance hypertension management in older people is the uncertainty about the most appropriate blood pressure targets in terms of benefits and risks. The latest available guidelines for hypertension management in the elderly were mainly based on the same body of evidence but differ significantly in target systolic blood pressure (SBP) values [3, 4]. For example, the initiation of antihypertensive therapy is ≥ 140/90 mmHg for elderly patients (65 ~ 79 years), ≥ 160/90 mmHg for patients older than 80 years according to the European Society of Cardiology / European Society of Hypertension 2018 Guidelines [5], while the 2019 Chinese guideline recommends to start the drug treatment when blood pressure is ≥ 140/90 mmHg for patients aged around 65 ~ 79 years and ≥ 150/90 mmHg for patients aged ≥ 80 years [4]. The American College of Physicians / American Academy of Family Physicians 2017 Guideline recommends that the threshold for antihypertensive therapy is SBP ≥ 150 mmHg for patients older than 60 years [6]. What’s more, the American Heart Association / American College of Cardiology 2017 Guideline suggests to initiate the antihypertensive therapy for any patient older than 65 years as long as his/her blood pressure higher than 130/80 mmHg [7]. Together with the controversial association of lowering SBP with all-cause mortality among oldest-old (older than 80 years) [8, 9], these factors have further fueled debate. A better understanding of the risks conferred by SBP control is needed to direct clinical decisions and to prevent either excess or inadequate use of antihypertensive treatments in the elderly population [10].

Besides, blood pressure variability was identified as a potential risk factor for adverse outcomes, such as arterial remodeling [11], macro- and micro-vascular disease [12], and mortality [12, 13]. Study conducted among Chinese hypertensive adults found that SBP variability (SBPV) has significant prognostic value, in addition to baseline SBP for the risk of cardiovascular disease [12]. A meta-analysis published in 2016 also reported that long-term SBPV is associated with cardiovascular and mortality outcomes, over and above the effect of mean SBP [13]. However, to our knowledge, the link between SBPV and mortality has not previously been specifically investigated in elderly people, especially among octogenarians or nonagenarians.

Using data from the Chinese Longitudinal Healthy Longevity Survey (CLHLS), we aimed to investigate the association between both mean SBP and SBPV and all-cause mortality in Chinese oldest-old.

## Methods

### Data source and study population

This study is embedded in the CLHLS, which is a national cohort focusing on older Chinese people and is the largest cohort of centenarians in the world. A detailed study design of CLHLS has been published elsewhere [14]. By using the multistage cluster sampling approach, all centenarians living in the sampled community or village were invited, with their 1:1 matched octogenarian and nonagenarian who living in the same area. The CLHLS study was approved by the Research Ethics Committee of Peking University (IRB00001052-13074), and all participants or their proxy respondents provided written informed consent.

The baseline survey of the current study was conducted in 2005, with follow-up waves conducted in 2008, 2011, 2014, and 2018. A further extension of the cohort was initiated in the 2008 and 2011 waves following the same study protocol. An overview of the study population is shown in **e-Fig. 1**.

### Exposures and outcome

As shown in **e-Fig. 2**, the primary exposures were mean SBP and SBPV, measured between baseline and second wave. The primary outcome was all-cause mortality, identified from the second wave.

Mean SBP was assessed by calculating the updated arithmetic mean of SBP in the consecutive two waves from 2005 onwards ((Second wave + Baseline)/2). Within-individual SBPV between two sequential waves was defined as the difference in SBP between two waves divided by the mean ((Second wave-Baseline)/mean). To account for slightly different visit intervals, this measurement was further scaled to the average variation per year, assuming a constant rate of variation between the two waves [15].

### Covariates

Information on covariates was collected at baseline using a structured questionnaire, including sociodemographic characteristics (body mass index (BMI), educational level, economic income (high vs. medium/low)), lifestyle habits and medical history. Smoking status was defined as current, past, or never smoker. Alcohol consumption (current vs. former/never) was assessed based on the question “Do you currently drink alcohol?”. Visual status was defined as “good” or “poor” according to whether participants could identify the break in the image of a circle held before them. Cognitive function was measured by the Chinese version of Mini-Mental State Examination (MMSE) and we defined cognitive impairment based on both MMSE score and education level: <18 for those without formal education, < 21 for those with 1–6 years of education, < 25 for those with more than 6 years of education [16]. Restriction in daily living activities was defined as a participant being dependent on toileting, bathing, indoor activities, dressing, eating, or continence. Comorbidity was defined according to the number of the self-reported disease, including diabetes mellitus, cardiovascular disease, stroke, respiratory disease, and cancer.

Frailty was assessed by the adjusted osteoporotic fracture index [17, 18], which including three components: (1) underweight (BMI < 18.5 kg/m^2^); (2) participants having trouble standing up from a chair without the assistance of arms; and (3) a positive response to the question “how many times suffering from serious illness in the past two years”. We categorized frailty status into: frail (two or three components), pre-frail (one component), and robust (no component).

### Statistical methods

**Primary analyses.** Our analysis focused on the association between changes in SBP (mean SBP and SBPV), assessed over two sequential study waves, and all-cause mortality, among oldest-old. Person-time accumulated from the second wave (first assessment of mean SBP and SBPV) until the date of death, date of loss to follow-up, or the end date of follow-up (the latest follow-up visits for the CLHLS conducted in 2018), whichever came first.

We first investigated the associations between continuous mean SBP and SBPV and all-cause mortality using Cox proportional hazards models with penalized splines, which examine the potential non-linear or irregular shape of the hazard functions. Other covariates, such as BMI, could also exert a non-linear effect here. Following the suggested procedure [19], we obtained the corresponding multivariable degree of freedom based on the corrected Akaike information criterion and biological plausibility. Then, we stratified mean SBP and SBPV into quintile with the reference group defined based on non-linear associations we found above. Briefly, mean SBP was categorized into five groups: “<122 mmHg”, “122 ~ 130 mmHg”, “130 ~ 138 mmHg”, “138 ~ 148 mmHg”, and “>148 mmHg” with “138 ~ 148 mmHg” used as refer. SBPV was also categorized into quintile: “<-4.9 %/year”, “-4.9~-1.4 %/year”, “-1.4 ~ 1.8 %/year”, “1.8 ~ 5.4 %/year”, and “>5.4 %/year” with middle quintile “-1.4 ~ 1.8 %/year” used as refer.

All Cox models were adjusted for baseline covariates, including age, sex, BMI, educational background, economic income, smoking status, alcohol consumption, visual status, cognitive impairment, restriction in activities of daily living, comorbidity, and cohort. The proportional hazard assumption was assessed by visual inspection of the scaled Schoenfeld residuals plot. Single imputation with the expectation-maximization method was used to deal with missing covariates (percentage of missing covariates: 10.4 %).

**Additional analyses.** To identify potential effect modification, we stratified the analyses by self-reported doctor-diagnosed hypertension and frailty status at baseline. Interaction was formally tested on a multiplicative scale by adding a product term to the model.

**Sensitivity analyses**. Given the previously reported terminal decline in SBP at the end-of-life [20], we checked the potential impact of reverse causality by repeating the main analyses using 1-year and 2-year lag periods, separately. We also conducted the complete case analyses taking into account the uncertainty of imputed values.

## Results

### Participant characteristics

Of the 6639 participants included (cohort 2005: 3754, cohort 2008: 2428, cohort 2011: 457), 4018 (61 %) were women, and the mean (SD) age was 90.7 (7.0) years. Table 1 describes the characteristics for participants. During a median follow-up of 2.4 years (interquartile range 1.4–4.8), 4622 died among 6639 included participants (overall mortality rate 215.5 cases per 1000 person-years).

**Table 1:** Characteristics of the included participants

	Total population (*n* = 6639)	Cohort 2005 (*n* = 3754)	Cohort 2008 (*n* = 2428)	Cohort 2011 (*n* = 457)
Age, year	90.7 (7.0)	90.1 (6.7)	91.1 (7.2)	92.5 (8.2)
Sex, female	4018 (61 %)	2243 (60 %)	1472 (61 %)	303 (66 %)
Body mass index, kg/m^2^	19.4 (3.3)	18.8 (3.2)	20.0 (3.3)	20.7 (3.6)
Education, illiterate	4621 (70 %)	2547 (68 %)	1723 (71 %)	351 (77 %)
Residence				
city	1213 (18 %)	814 (22 %)	381 (16 %)	18 (4 %)
town	1260 (19 %)	757 (20 %)	480 (20 %)	23 (5 %)
rural	4166 (63 %)	2183 (58 %)	1567 (65 %)	416 (91 %)
Economic income				
median/low	5561 (84 %)	3100 (83 %)	2079 (86 %)	382 (84 %)
high	1078 (16 %)	654 (17 %)	349 (14 %)	75 (16 %)
Smoke				
current	1057 (16 %)	642 (17 %)	372 (15 %)	43 (9 %)
past	890 (13 %)	572 (15 %)	275 (11 %)	43 (9 %)
never	4692 (71 %)	2540 (68 %)	1781 (73 %)	371 (81 %)
Current drinker	1261 (19 %)	754 (20 %)	433 (18 %)	74 (16 %)
Cognitive impairment	1620 (24 %)	866 (23 %)	632 (26 %)	122 (27 %)
Restriction on activities of daily living	1258 (19 %)	772 (21 %)	379 (16 %)	107 (23 %)
Poor visual function	2546 (38 %)	1450 (39 %)	915 (38 %)	181 (40 %)
Frailty status				
Robust	2255 (34 %)	1128 (30 %)	952 (40 %)	175 (42 %)
Pre-frailty	2830 (43 %)	1633 (44 %)	1012 (42 %)	185 (44 %)
Frailty	1466 (22 %)	978 (26 %)	430 (18 %)	58 (14 %)
Self-reported doctor-diagnosed hypertension	1029 (17 %)	550 (16 %)	366 (16 %)	113 (25 %)
Diabetes mellitus	102 (2 %)	59 (2 %)	34 (1 %)	9 (2 %)
Cardiovascular disease	487 (7 %)	292 (8 %)	163 (7 %)	32 (7 %)
Stroke and cerebrovascular disease	292 (4 %)	149 (4 %)	106 (4 %)	37 (8 %)
Respiratory disease	725 (11 %)	469 (12 %)	218 (9 %)	38 (8 %)
Cancer	17 (0 %)	9 (0 %)	6 (0 %)	2 (0 %)
Comorbidity				
0	5258 (79 %)	2932 (78 %)	1971 (81 %)	355 (78 %)
1	1165 (18 %)	682 (18 %)	395 (16 %)	88 (19 %)
>=2	216 (3 %)	140 (4 %)	62 (3 %)	14 (3 %)
Interval (baseline ~ second), year	3.1 (0.3)	3.2 (0.1)	3.1 (0.3)	2.0 (0.1)
**Baseline wave**				
Systolic blood pressure, mmHg	134.7 (20.2)	130.8 (17.9)	139.3 (21.6)	142.0 (22.3)
Diastolic blood pressure, mmHg	80.6 (11.6)	82.1 (11.6)	78.6 (11.2)	79.7 (11.7)
**Second wave**				
Systolic blood pressure, mmHg	135.8 (21.9)	134.8 (21.3)	136.4 (22.1)	141.6 (24.5)
Diastolic blood pressure, mmHg	78.9 (12.2)	78.7 (12.2)	79.2 (12.2)	78.9 (12.3)

Note: Data are mean (standard deviation) for continuous variables, n (%) for categorized variables

### Mean of systolic blood pressure and all-cause mortality

**Fig. 1:**
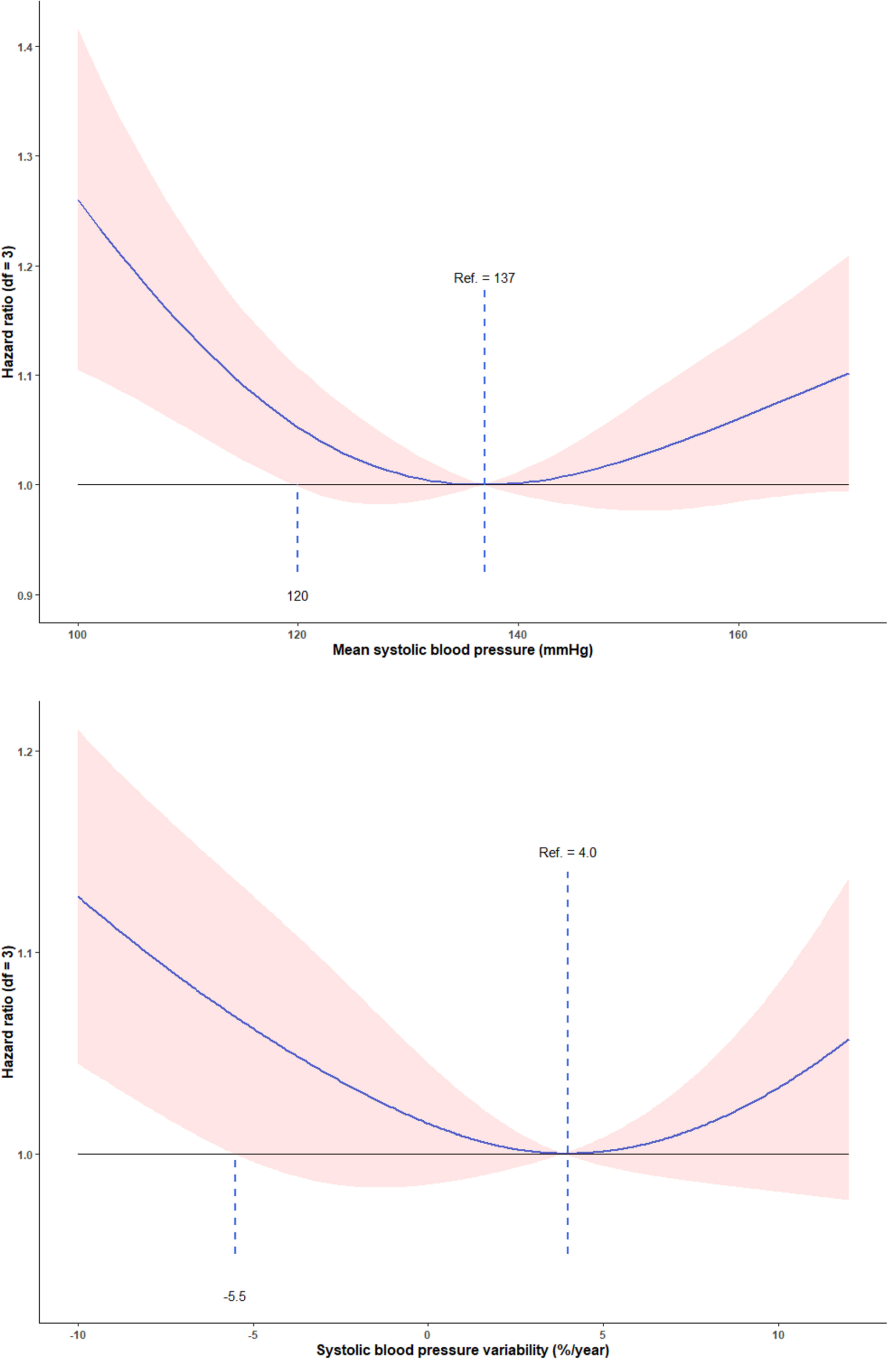
Associations of mean systolic blood pressure and systolic blood pressure variability and all-cause mortality. Note: Adjusted for age, sex, body mass index, educational background, economic income, smoking status, alcohol consumption, visual status, cognitive impairment, restriction in activities of daily living, comorbidity, and cohort at baseline.

**Fig. 2:**
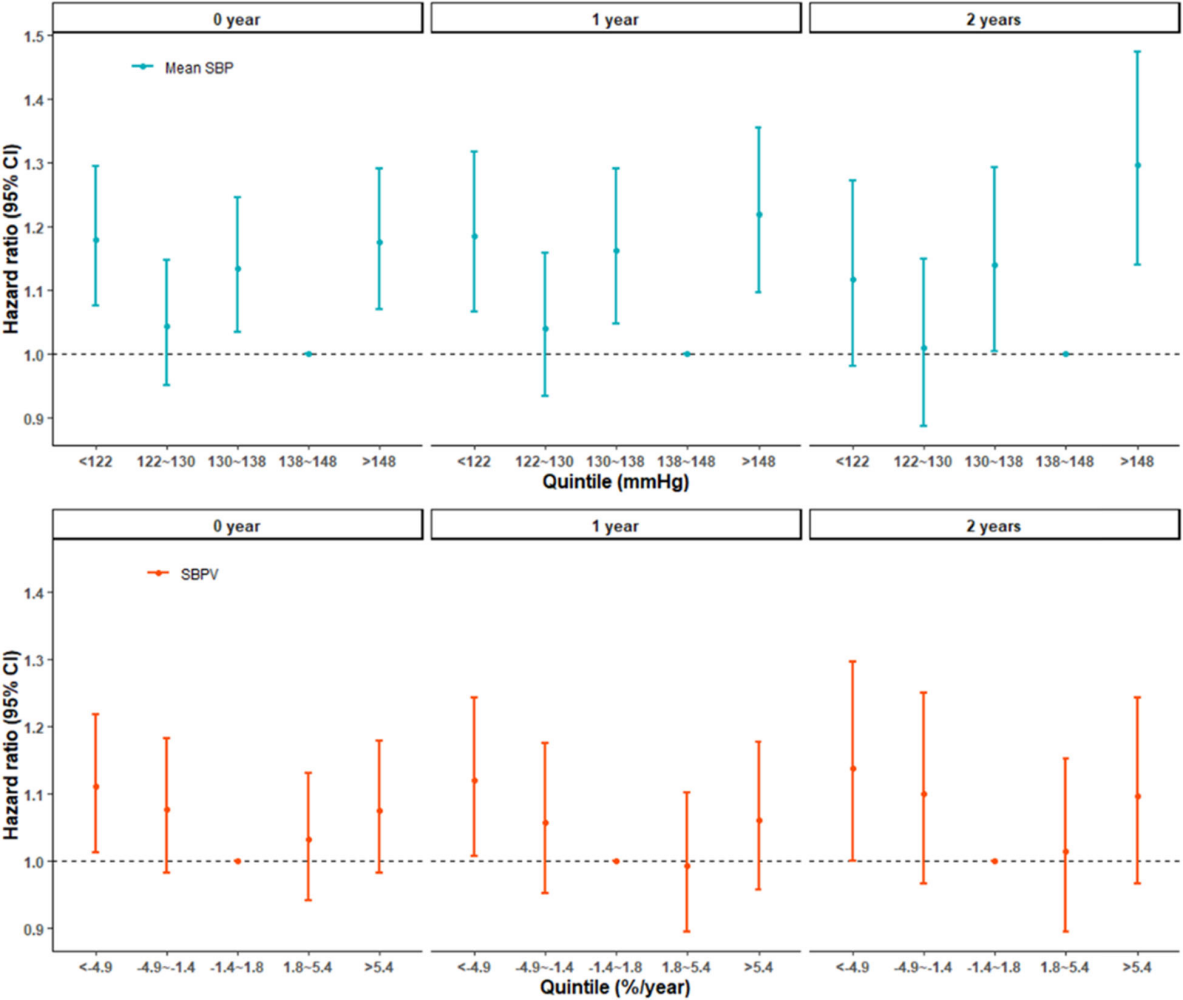
Associations of categorized mean systolic blood pressure (mean SBP) and systolic blood pressure variability (SBPV) and all-cause mortality, using different lag periods. Note: Hazard ratios were adjusted for age, sex, body mass index, educational background, economic income, smoking status, alcohol consumption, visual status, cognitive impairment, restriction in activities of daily living, comorbidity, and cohort at baseline.

The results of the Cox proportional hazards model with penalized splines suggested a U-shaped association between mean SBP and all-cause mortality (Fig. 1). Over 2.4 (median) years, the mean SBP value that conferred the minimum mortality risk was 137 mmHg. Table 2**and** Fig. 2 show the hazard ratios (HRs) and 95 % confident intervals (CIs) of mortality by quintiles of mean SBP. Compared to participants with mean SBP of 138 ~ 148 mmHg, those among the lowest (< 122 mmHg) or highest (> 148 mmHg) quintile had a higher risk of all-cause mortality, with HR 1.18 (95 %CI: 1.08, 1.29), and 1.18 (95 % CI: 1.07, 1.29), respectively.

**Table 2:** Associations of categorized mean systolic blood pressure (mean SBP) and systolic blood pressure variability (SBPV) and all-cause mortality, using different lag periods

Variables	Lag periods (years)	Hazard ratios (95 % CI) ^a^				
		**Q1 (< 122 mmHg)**	**Q2 (122 ~ 130 mmHg)**	**Q3 (130 ~ 138 mmHg)**	**Q4 (138 ~ 148 mmHg)**	**Q5 (> 148 mmHg)**
Mean systolic blood pressure	0	1.18 (1.08, 1.29)	1.04 (0.92, 1.15)	1.14 (1.04, 1.24)	1	1.18 (1.07, 1.29)
	1	1.19 (1.07, 1.32)	1.04 (0.93, 1.16)	1.16 (1.05, 1.29)	1	1.22 (1.10, 1.35)
	2	1.12 (0.98, 1.27)	1.01 (0.89, 1.15)	1.14 (1.00, 1.29)	1	1.30 (1.14, 1.47)
		**Q1 (<-4.9 %/year)**	**Q2 (-4.9~-1.4 %/year)**	**Q3 (-1.4 ~ 1.8 %/year)**	**Q4 (1.8 ~ 5.4 %/year)**	**Q5 (> 5.4 %/year)**
Systolic blood pressure variability	0	1.11 (1.01, 1.22)	1.08 (0.98, 1.18)	1	1.03 (0.94, 1.13)	1.08 (0.98, 1.18)
	1	1.12 (1.01, 1.24)	1.06 (0.95, 1.17)	1	0.99 (0.89, 1.10)	1.06 (0.96, 1.18)
	2	1.14 (1.00, 1.30)	1.10 (0.97, 1.25)	1	1.02 (0.89, 1.15)	1.10 (0.97, 1.24)

^a^ With adjustment for age, sex, body mass index, educational background, economic income, smoking status, alcohol consumption, visual status, cognitive impairment, restriction in activities of daily living, comorbidity, and cohort at baseline

**Fig. 3:**
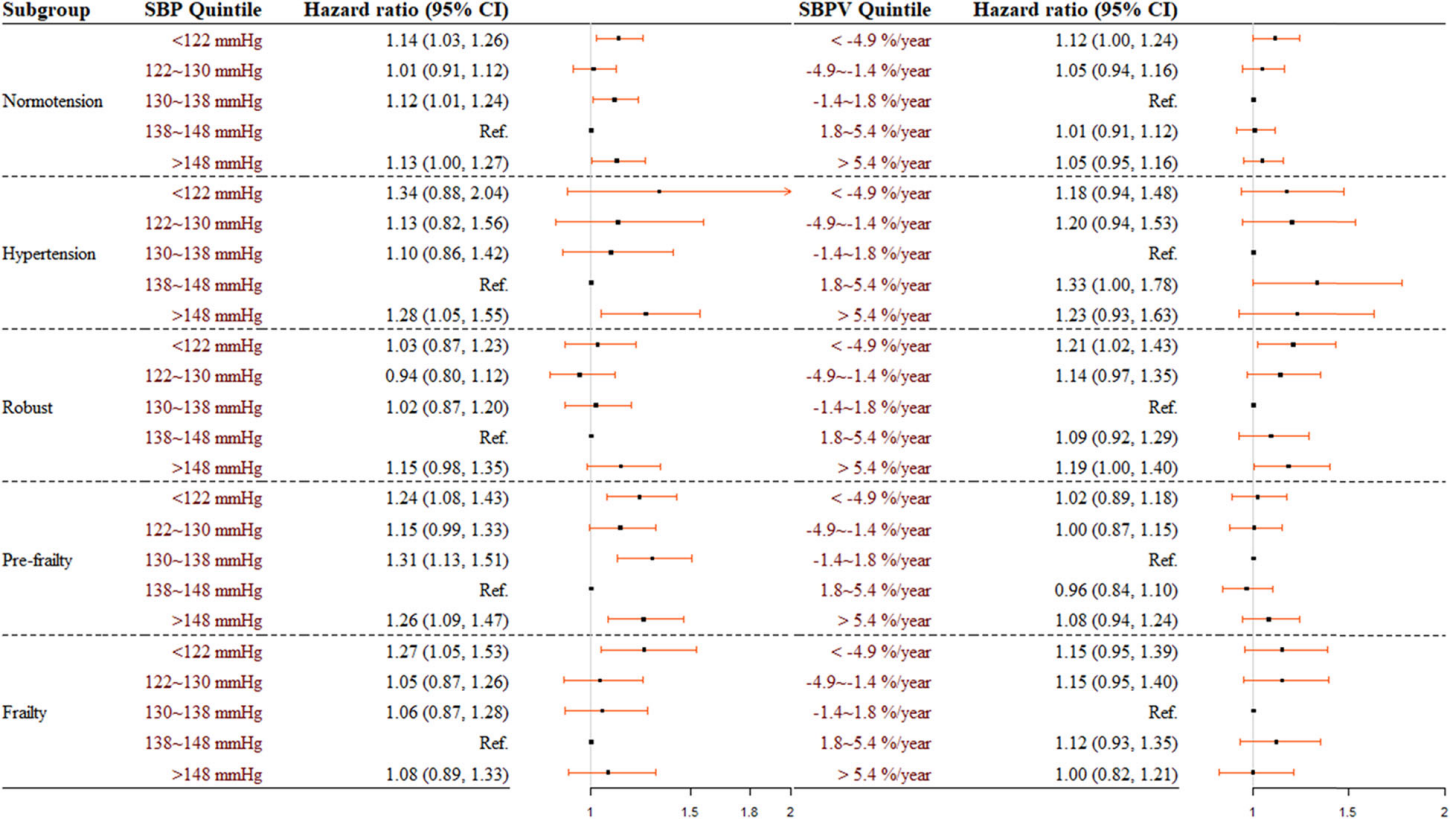
Associations of categorized mean systolic blood pressure (mean SBP) and systolic blood pressure variability (SBPV) and all-cause mortality among different subgroups. Note: Hazard ratios were adjusted for age, sex, body mass index, educational background, economic income, smoking status, alcohol consumption, visual status, cognitive impairment, restriction in activities of daily living, comorbidity, and cohort at baseline.

Although no significant interaction was found during additional analyses, a stronger association between mean SBP and mortality was noted among those who had self-reported hypertension or with pre-frailty status (Fig. 3). Among pre-frailty individuals, compared to those with mean SBP of 138 ~ 148 mmHg, participants among the lowest (< 122 mmHg) or highest (> 148 mmHg) quintile had a significant higher mortality risk, with HR 1.24 (95 %CI: 1.08, 1.43), and 1.26 (95 % CI: 1.09, 1.47), respectively.

### Systolic blood pressure variability and all-cause mortality

Cox proportional hazards models with penalized splines shown a U-shaped association between SBPV and all-cause mortality; the lowest mortality risk was found among participants with SBPV of 4.0 %/year (Fig. 1). The associations of a larger SBP variation with an increased mortality risk were observed for both rises and large falls in SBP after categorizing SBPV by quintile (Table 2; Fig. 2**)**. The HR was 1.11 (comparing lowest versus middle quintile; 95 % CI: 1.01, 1.22) with large falls in SBPV and 1.08 (comparing highest versus middle quintile; 95 % CI: 0.98, 1.18) with large rises in SBPV.

We found no significant interaction term during additional analyses. After further stratifying participants by hypertension or frailty status, similar trends were found in each subgroup, while the magnitude of association with SBPV was somewhat larger among participants with self-reported hypertension (Fig. 3).

### Sensitivity analyses

Findings were consistent in the sensitivity analyses (see Fig. 2 and **e-Fig. 3**). These association estimates appeared to have wider CIs after different lag periods were considered but still suggested U-shaped. Results based on data from complete case also did not affect the main finding.

## Discussion

Based on a large-scale community-based cohort, our results indicated U-shaped associations between late-life SBP and SBPV and risk of all-cause mortality, with mean SBP of 137 mmHg, SBPV of 4.0 %/year, related to the lowest mortality risk. The associations of a larger SBP variation with an increased mortality risk were observed for both rises and large falls in SBP. Consistent with the 2019 Chinese guideline which recommends that the threshold for antihypertensive therapy is SBP ≥ 150 mmHg for patients older than 80 years, our findings further added this by suggesting that keeping a stable blood pressure level is also important for hypertension management among the oldest-old.

### Systolic blood pressure and mortality

The observed association between lower SBP and increased risk of all-cause mortality among oldest-old is in line with previous concern regarding the intensity of antihypertensive treatment in elderly population [21]. Although low blood pressure per se causes harm, it also could be an indicator of poor health status. Low SBP was associated with mortality even in fit participants [20]. Results from the Berlin Initiative Study found that control blood pressure below 140/90 mmHg during antihypertensive treatment still associated to an increased risk of mortality in participants ≥ 70 years [22]. In contrast, the link between higher SBP and mortality has been consistent in older age with the only question regarding the detailed treatment strategy, such as the definition of ‘old’ patients, the definition of arterial hypertension, blood pressure target value in older patients [3]. A previous study also based on the CLHLS cohort reported a U-shaped association of mortality with SBP, with lower risk among participants who have a middle range of SBP (107 ~ 154 mmHg) [18]. Unlike their study which only considered single assessment of blood pressure, we investigated changes of blood pressure during three years interval. Our findings, therefore, extend their evidence by demonstrating that keeping a long-term stable SBP level is also important for lowering the mortality risk in oldest-old.

A U-shaped or J-shaped relationship between targeted SBP and risk of morbidity and mortality has long been suggested [4]. This hypothesis is mainly based on a presumed SBP threshold for organ blood flow autoregulation, and the potential role of blood pressure as a compensatory mechanism for preserving organ function [23]. Considering the totality of evidence, less aggressive treatment would be an optimal approach in treating hypertension in older people [24].

### Systolic blood pressure variability and mortality

Variability in blood pressure has been recognized as a potential risk factor, whereas no standard formula available for its calculation. Multiple measures, such as standard deviation, coefficient of variation, and root successive variance, hamper the understanding of blood pressure variability [13]. In our study, to adjust for mean blood pressure and account for different visit intervals, the blood pressure variability was calculated as the difference in SBP between two waves divided by the mean and further scaled into the average variation per year. Using the same measure, Yuan et al. reported that a large blood pressure variation over years was associated with changes of subclinical brain structural [25] and an increased long-term risk of dementia [15]. Here we found an elevated risk of all-cause mortality with a large rise and fall in later-life SBPV among oldest-old people.

Blood pressure variability, especially long-term, is associated with cardiovascular and mortality outcomes and shows additional prognostic value independent of mean blood pressure [13]. Although the underline mechanism has not been well understood, it could be partly explained by arterial stiffness [26] or the changes to antihypertensive drugs resulting from poor blood pressure control [27]. Greater blood pressure variability also decreases endothelial function [28] and leads to greater cardiac and vascular damage as well as progression of the left ventricular mass index [29][32]. Future studies should explore this relationship in-depth to determine dynamic and individualized targets for older people.

### Strengths and limitations

Our study has some unique and useful features. The most important feature is that based on the large-scale population-based cohort, we thoroughly investigated associations of changes of late-life SBP with risk of mortality among oldest-old, which filled in a certain knowledge gap about control of late-life blood pressure in older population [30]. On the other hand, there are still some limitations in our study. Firstly, although we have carefully adjusted many potential confounders, other unknown factors were still possible. Many factors, such as treatment of hypertension, blood glucose, were not collected in the CLHLS and could not be analyzed here. Secondly, frailty index used in our study was adapted from a former study [18] and has not been further validated. Considering frailty status is an important confounder for assessing health effects of hypertension among elderly population [31, 32], our results should be interpreted with caution and other studies with validated frailty measurement are needed. Finally, our study has a roughly 3-year run-in period to meet the requirement for calculating the primary exposure suggesting that the included participants could be selected healthy individuals, limiting the generalizability of our findings to the general population.

## Conclusions

Our findings suggest that SBP variability might be an important factor in understanding mortality risk in oldest-old, affirming the need to develop better strategies for blood pressure management in this population.

## Supplementary information

**Additional file 1.**

## Data Availability

The original CLHLS dataset are available at https://opendata.pku.edu.cn/dataverse/CHADS. The full dataset used in this analysis are available from the corresponding author upon reasonable request.

## Abbreviations

CLHLS: the Chinese Longitudinal Healthy Longevity Survey
SBP: systolic blood pressure
SBPV: systolic blood pressure variability
MMSE: Mini-Mental State Examination
BMI: body mass index
HR: hazards ratio
