# Supplementary material for: Changes in late-life systolic blood pressure and all-cause mortality among oldest-old people in China: the chinese longitudinal healthy longevity survey
Source: BMC Geriatr. 2021 Oct 18;21:562. doi: 10.1186/s12877-021-02492-4 (PMC8522233; doi:10.1186/s12877-021-02492-4)
Supplement: Supplementary file 1 — Additional file 1. [file 12877_2021_2492_MOESM1_ESM.docx]

**Changes in late-life systolic blood pressure and all-cause mortality among oldest-old people in China: the Chinese Longitudinal Healthy Longevity Survey**

**Supplement material**

**e-Figure 1**. Flow chart of the study population.

**e-Figure 2**. Schematic diagram of the analyses relating control of systolic blood pressure to risk of all-cause mortality at different cohorts.

**e-Figure 3**. Associations of categorized mean systolic blood pressure (mean SBP) and systolic blood pressure variability (SBPV) and all-cause mortality, using different lag periods during complete case analyses.

**e-Figure 1**. Flow chart of the study population.


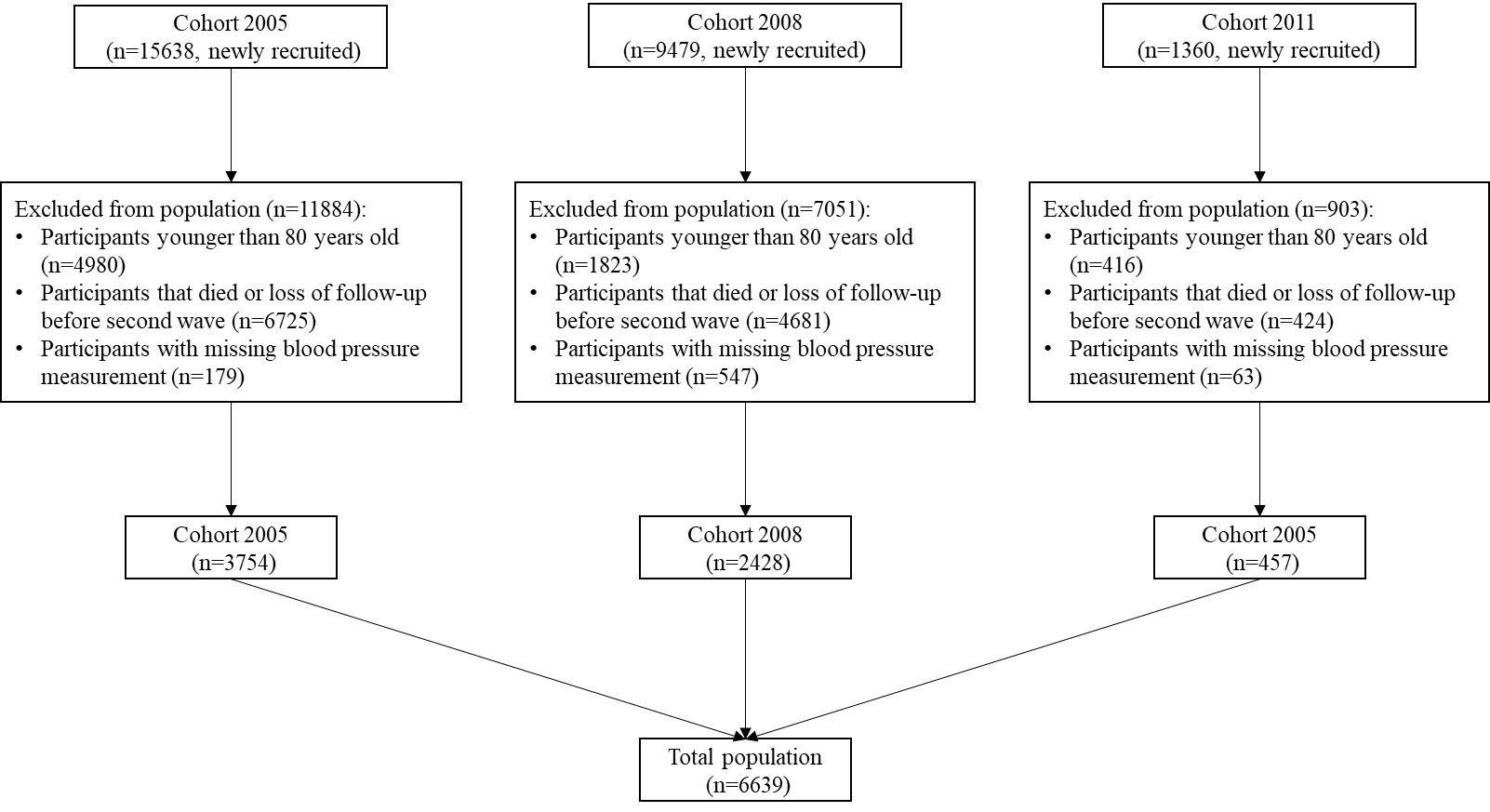


**e-Figure 2**. Schematic diagram of the analyses relating control of systolic blood pressure to risk of all-cause mortality at different cohorts.

**
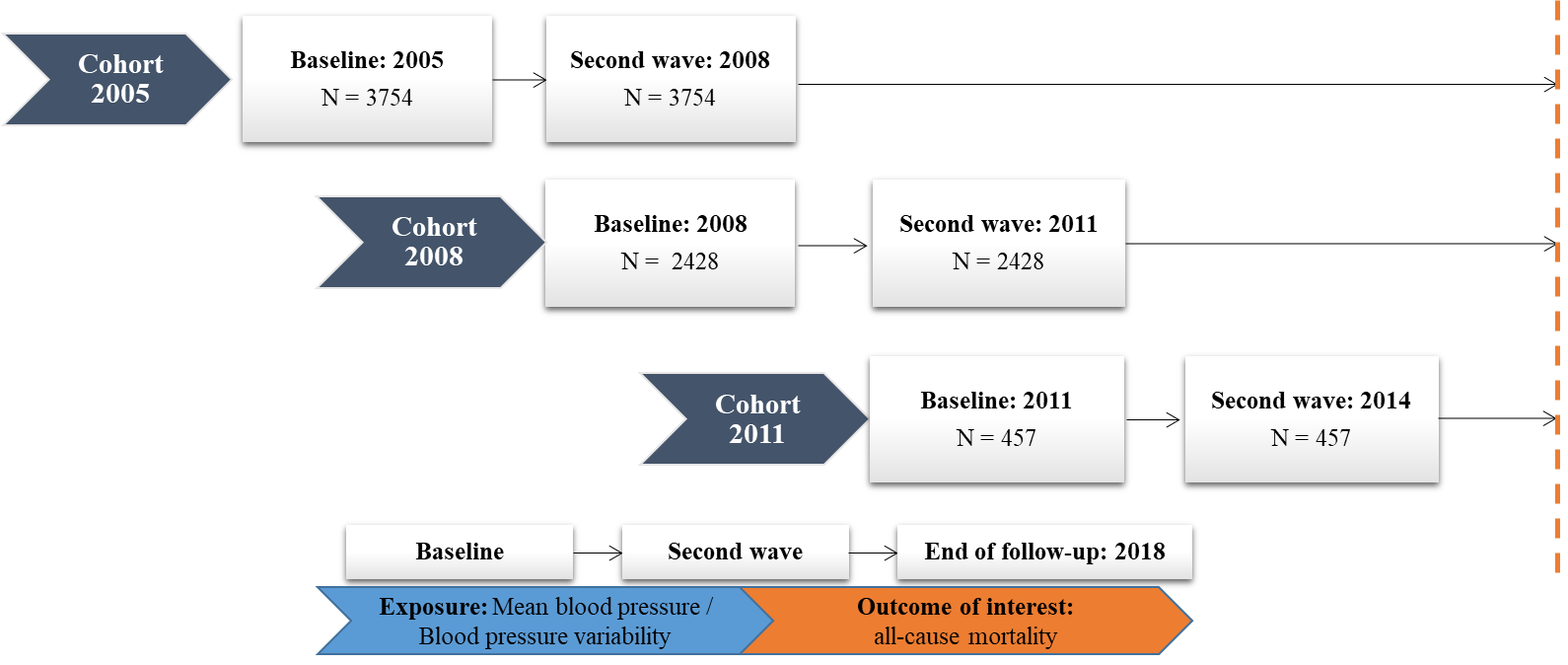
**

**e-Figure 3**. Associations of categorized mean systolic blood pressure (mean SBP) and systolic blood pressure variability (SBPV) and all-cause mortality, using different lag periods during complete case analyses (n=5951).


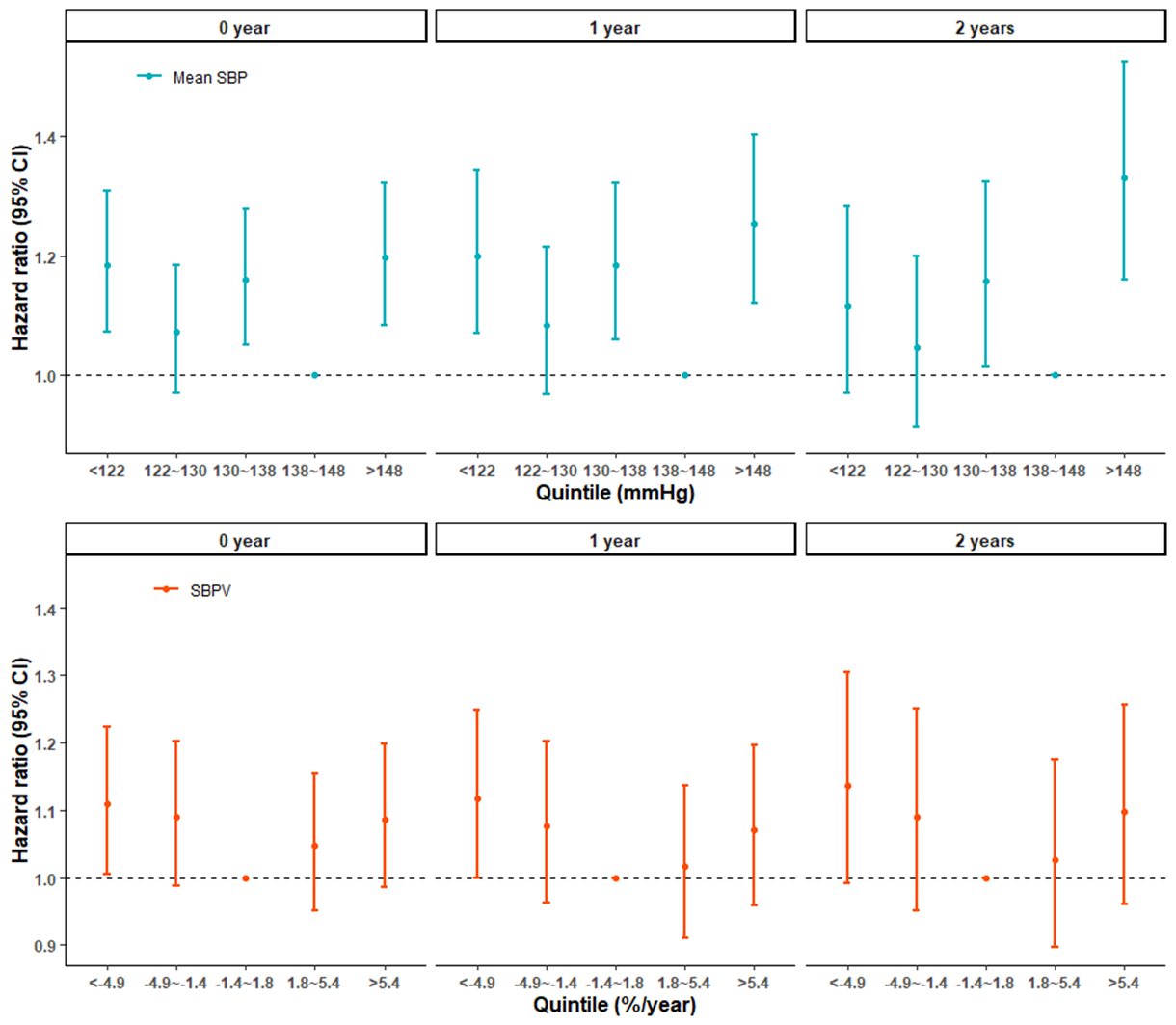


**Note**. Hazard ratios were adjusted for age, sex, body mass index, educational background, economic income, smoking status, alcohol consumption, visual status, mild cognitive impairment, restriction in activities of daily living, comorbidity, and cohort at baseline.
